# Impact of Gas–Solid Reaction Thermodynamics on the Performance of a Chemical Looping Ammonia Synthesis Process

**DOI:** 10.1021/acs.energyfuels.2c01372

**Published:** 2022-07-01

**Authors:** Reinaldo Juan Lee Pereira, Wenting Hu, Ian S. Metcalfe

**Affiliations:** School of Engineering, Newcastle University, Newcastle upon Tyne NE1 7RU, United Kingdom

## Abstract

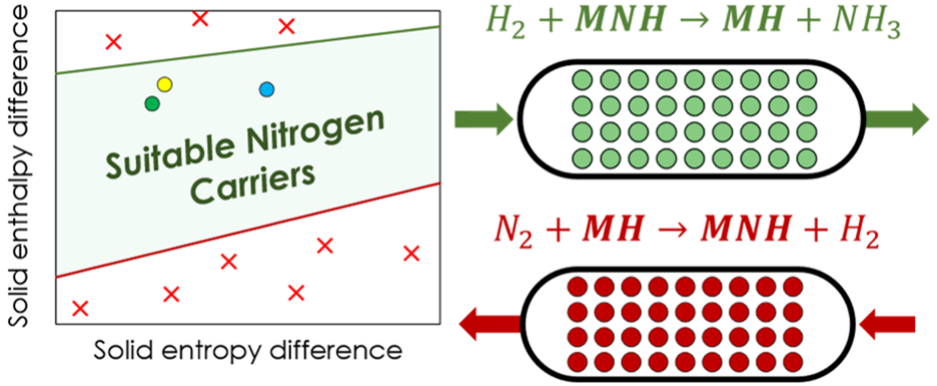

Novel ammonia catalysts seek to achieve high reaction rates under milder conditions, which translate into lower costs and energy requirements. Alkali and alkaline earth metal hydrides have been shown to possess such favorable kinetics when employed in a chemical looping process. The materials act as nitrogen carriers and form ammonia by alternating between pure nitrogen and hydrogen feeds in a two-stage chemical looping reaction. However, the thermodynamics of the novel reaction route in question are only partially available. Here, a chemical looping process was designed and simulated to evaluate the sensitivity of the energy and economic performance of the processes toward the appropriate gas–solid reaction thermodynamics. Thermodynamic parameters, such as reaction pressure and especially equilibrium ammonia yields, influenced the performance of the system. In comparison to a commercial ammonia synthesis unit with a 28% yield at 150 bar, the chemical looping process requires a yield greater than 38% to achieve similar energy consumptions and a yield greater than 26% to achieve similar costs at a given temperature and 150 bar. Entropies and enthalpies of formation of the following pairs were estimated and compared: LiH/Li_2_NH, MgH_2_/MgNH, CaH_2_/CaNH, SrH_2_/SrNH, and BaH_2_/BaNH. Only the LiH/Li_2_NH pair has satisfied the given criteria, and initial estimates suggest that a 62% yield is obtainable.

## 1 Introduction

The Haber–Bosch process enabled large-scale fertilizer production, a key driver in human population growth.^1^ At present, 70% of ammonia is used to produce synthetic nitrogen fertilizers, which are estimated to feed half of the world’s population.^1,2^ Although vital to human life, 96% of ammonia is currently produced using coal and natural gas.^2^ Consequently, its production accounts for 2% of the world’s energy consumptions and 1.3% of global greenhouse gas emissions.^2^ From a social, environmental, and economic point of view, there are incentives to eliminate greenhouse gas emissions and/or reduce its energy consumptions.

Improvements in ammonia catalysts over the last century have sought to decrease energy consumption and economic cost of the process. Initial formulations based on iron catalysts synthesized ammonia at 300 bar and 350–500 °C, which incurred high compression duties and costs.^3^ Iron catalysts have retained their commercial success and progressed from promoted magnetite (K_2_O–Fe_3_O_4_) to wüstite (K_2_O–Fe_1–*x*_O) based catalysts, which synthesize ammonia at 120–150 bar and 350–500 °C. Catalyst formulations based on ruthenium have also been commercialized and can synthesize ammonia at 90 bar and 325–450 °C. Although the equilibrium conversion is favored by high pressures and low temperatures (reaction R1), high temperatures are required to be kinetically active. Therefore, succeeding catalyst formulations have aimed to achieve increased rates of reaction at lower temperatures and pressures.
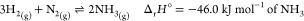
R1The Sabatier principle suggests that interactions between the catalyst and reactant should be neither too strong nor too weak. A quantitative approach to this principle has demonstrated that the ammonia synthesis rates of transition metal catalysts are limited by a linear scaling relationship between the adsorption energy and activation energy of nitrogen.^4^ In addition, effects such as hydrogen inhibition and oxygen poisoning are also important.^5,6^ Therefore, interactions with all three species need to be considered in the design of an active ammonia catalyst. Novel materials and reaction routes have been explored to overcome these rate limitations. Materials such as electrides,^7−9^ hydrides,^10−15^ nitrides,^16−19^ and oxides^20,21^ have shown to improve synthesis rates under milder conditions by acting as co-catalysts or promoters for the transition metal catalysts. Alternatively, novel ammonia synthesis routes based on electrocatalytic, photocatalytic, plasma-catalytic, and chemical looping processes have the potential to produce ammonia using renewable energy sources or overcome the limitations of the conventional route.

Unlike conventional catalytic processes, chemical looping ammonia synthesis (CLAS) processes use nitrogen and hydrogen (or its precursors) in consecutive steps to form ammonia through a solid intermediate. A summary of known CLAS processes and their reaction conditions, feeds, and carrier materials are shown in Table 1. Among the routes using hydrogen and nitrogen as feeds, alkali/alkaline earth metal imide systems have been shown to successfully undergo multiple cycles of nitrogenation and hydrogenation to form ammonia.^22,23^ Few transition metal nitrides have been shown to undergo multiple cycles,^24^ and some have yet to show clear evidence of this.^25^ Ammonia can also be produced by reacting the nitrogen-carrying materials with water. The oxides produced from the hydrolysis reaction are reduced via hydrogen,^26^ carbon,^27^ methane,^28^ electricity,^29^ or light.^30^ This route can allow the steam reforming plant and ammonia synthesis unit to be replaced while achieving similar exergy efficiencies^31^ to the conventional process.^6^ However, forming ammonia in this manner requires separation of ammonia–water mixtures, which requires at least twice the power consumption for separation^32^ compared to the conventional process.^3^ The focus of this paper will be confined to the alkali/alkaline earth metal imide synthesis route., e.g., reactions R2a and R2b.
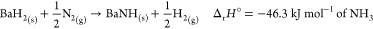
R2a
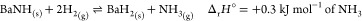
R2bWhen paired with transition metal catalysts, alkali and alkaline earth metal hydrides have been shown to overcome rate limitations as a result of scaling relations between the adsorption energy and activation energy of nitrogen^10^ and act as nitrogen carriers when employed in a chemical looping scheme.^22^ Ball-milling hydrides with transition metal catalysts (Ni, Co, Fe, Mn, Pd, and Cr) promote an otherwise inactive catalyst with the conventional^10,11^ and chemical looping^22,33,34,38^ routes. In some cases, the transition metal can also act as a nitrogen carrier (see Table 1). When nickel and barium hydride mixtures are supported on alumina, the ammonia synthesis rate at 300 °C and 1 bar (reaction R2) is roughly 3 times higher than the wüstite-based catalyst at 300 °C and 10 bar (reaction R1)^22^ and a half higher than the average rate of a two-bed reactor loaded with the wüstite catalyst at 350–480 °C and 110 bar (reaction R1).^3^ A comparison of ammonia synthesis rates with chemical looping and conventional catalysts is provided in section 1 of the Supporting Information .

**Table 1:** List of Known Chemical Looping Ammonia Processes and Their Operating Conditions^a^

CLAS material	*T* (°C)	*P* (bar)	feeds	reference
Alkali/Alkaline Earth Metal Imides				
Ni–BaNH	200–300	1	H_2_ and N_2_	(22)
Ni–BaNH/Al_2_O_3_	150–300	1		(22)
Mn_2_N–Li_2_NH	200–300	10		(33)
Mn_2_N–BaNH	200–300	1		(33)
Pd–Li_2_NH	200–300	1		(34)
Metal Nitrides				
Mn_6_N_2.58_	300–1000	1	H_2_ and N_2_	(26)
Ca_3_N_2_	300–1000	1		(26)
Sr_2_N	300–1000	1		(26)
Ni_3_ZnN	400–500	1		(25)
Mo_2_N	400–600	1		(24)
Metal Nitride Hydrolysis				
Mo_2_N	500–1000	1	H_2_O and H_2_ + N_2_	(28)
AlN	300–1000	1		(28)
Mn_4_N	300–1000	1		(28)
Cr_2_N	500–1000	1		(28)
Mg_3_N_2_	200–500	1		(28)
Zn_3_N_2_	300–1000	1		(28)
Ca_3_N_2_	200–500	1		(28)
Carbothermal Reduction and Hydrolysis				
AlN	800–1000	1	CH_4_ + N_2_ and H_2_O	(35)
TiO_2_–AlN	800–1000	1		(36)
ZrO_2_–AlN	800–1000	1		(37)
Electrocatalytic Reduction and Hydrolysis				
Li_3_N	25–100	1	electricity, H_2_O, and N_2_	(29)
Photocatalytic Reduction and Hydrolysis				
Mg_3_N_2_	>600	1	light + CH_4_, H_2_O, and N_2_	(30)

a The experimental conditions provided are for the ammonia synthesis step.

Both the kinetics and thermodynamics of this novel reaction route play a significant role in determining its feasibility and competitiveness. Although high ammonia synthesis rates are achievable under milder conditions with CLAS, the thermodynamics of reaction R2 differ from the conventional reaction (reaction R1), which can impact the cost of producing ammonia. For a given reaction scheme, such as reaction R2, the gas species, the reaction stoichiometry, and the gas phase thermodynamic properties are well-defined, whereas the solid phase can be varied. If suitable solid phase thermodynamic properties are present, the equilibrium conversion of either reaction R2a or R2b can be improved. However, the solid properties of many alkali and alkaline earth metal hydrides/imides are unknown; hence, it is unclear whether a more favorable equilibrium conversion can be achieved with this class of material, despite the improved kinetics. To date, only one study has investigated the impact of the reaction thermodynamics on the performance of CLAS.^39^

In this work, a new design for the CLAS unit is proposed and benchmarked against a conventional ammonia synthesis unit. The impact of the gas–solid reaction thermodynamics on the energy and economic costs of the CLAS unit was evaluated, and its sensitivity toward the equilibrium conditions of reactions was analyzed. The thermodynamic properties of the gas–solid reaction are assumed to satisfy a given equilibrium state for the sensitivity study. In doing so, favorable equilibrium conversions for the CLAS reaction can be defined, and the required solid properties may be benchmarked for the process. The properties of nitrogen carriers following the scheme of reaction R2 were estimated and compared to this benchmark to determine whether pairings with potentially better performance than the conventional process exist.

## 2 Methodology

### 2.1 Reference Case Simulation

**Figure 1:**
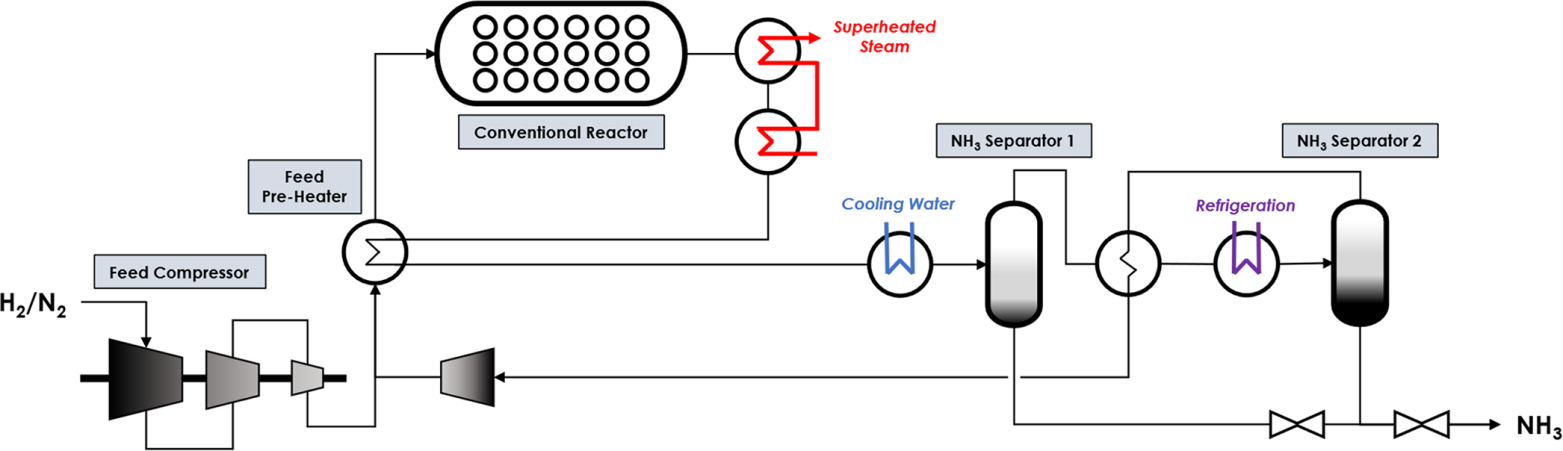
Process flow diagram for the reference ammonia synthesis unit (ref case).

A reference ammonia synthesis unit (ref case) with a 500 000 tonne per annum capacity was simulated in Aspen Plus to benchmark the chemical looping case. Conditions and configuration of the loop are based on commercial designs found in the literature.^3,6,40−42^ A process diagram for the synthesis loop is shown in Figure 1. The hydrogen feed is produced by steam methane reforming, and nitrogen is introduced via the secondary reformer. The feed is assumed to be at 20 °C and 25 bar and is compressed to 150 bar using a multi-stage compressor. The compressed feed is mixed with recycled gas, and the mixture is preheated to 340 °C using the hot gas exiting the reactor bed. Ammonia is then formed via an iron catalyst in a radial flow reactor. The product gas exits the reactor at 510 °C with an ammonia concentration of 16%.^3,40^ Heat within the product gas is used to generate steam at 125 bar and preheat the feed, and the remaining heat is removed using cooling water. Cooling the product gas partially condenses ammonia, which is then separated before refrigerating the remaining gas product to −30 °C. Ammonia condensed via refrigeration is separated, and the remaining gas is recompressed and recycled to the reactor. Gas purging does not need to be considered because the feed is assumed to contain pure gases. The product ammonia is depressurized to 20 bar for storage. See section 4 of the Supporting Information for the mass balance.

### 2.2 Chemical Looping Simulation

**Figure 2:**
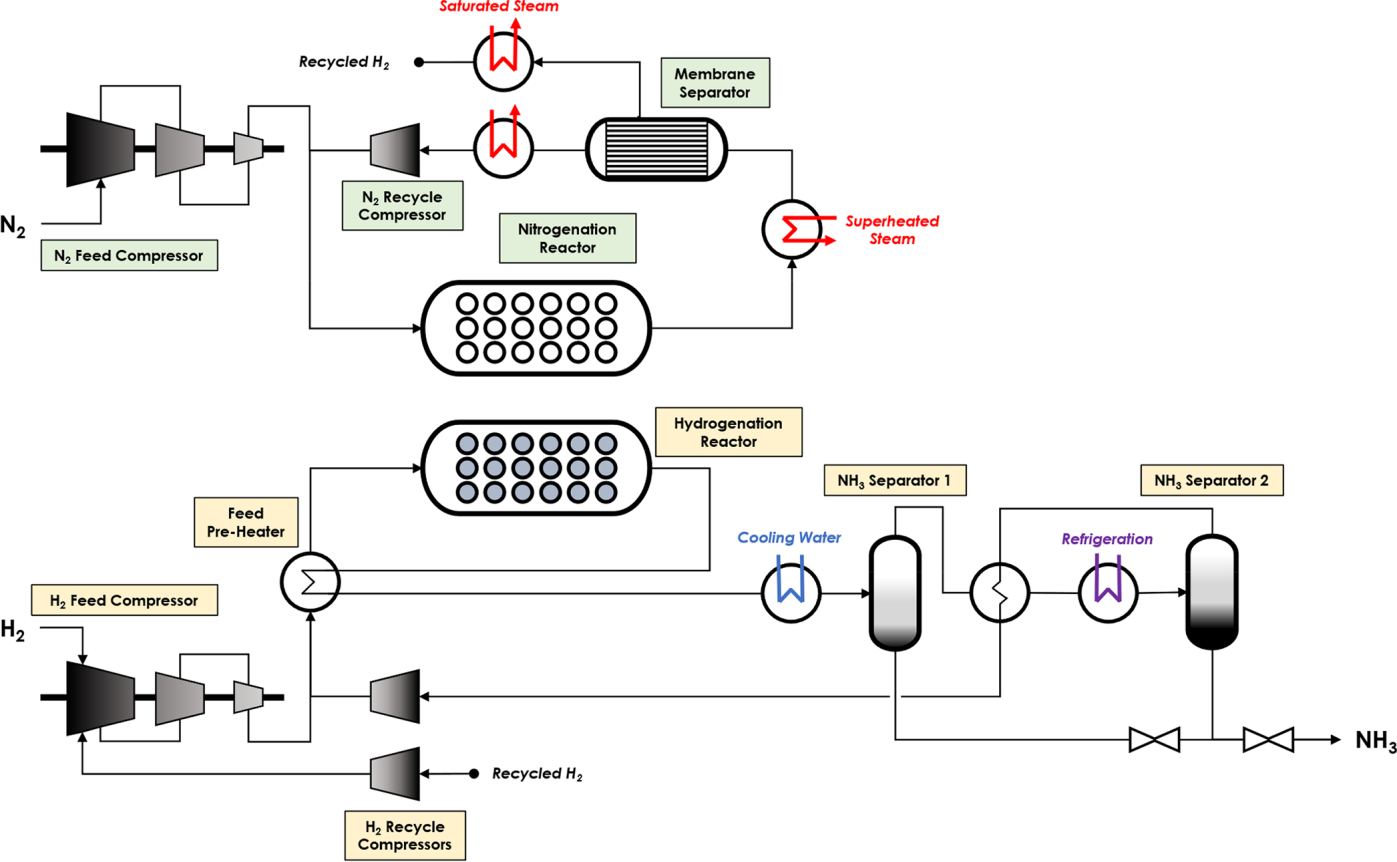
Process flow diagram for the chemical looping ammonia synthesis unit (CL case).

The chemical looping ammonia synthesis unit (CL case) comprises two distinct loops, as shown in Figure 2. The initial case is simulated such that the capacity, synthesis loop pressures, and ammonia yield are equal to that of the reference case. The configuration and conditions of the hydrogenation loop are identical to the reference case synthesis loop; however, further consideration is required for the configuration of the nitrogenation loop and the steam generation of the unit. Unlike the conventional process, separate feeds of nitrogen and hydrogen are required. Ammonia production processes, which produce nitrogen via air separation, can achieve this requirement with similar energy performances.^42,43^ The feeds are assumed to be at 20 °C and 25 bar and then compressed to 150 bar, mixed with their respective recycled streams, and preheated using the hot gases exiting the chemical looping reactors. See section 4 of the Supporting Information for the mass balance.

#### 2.2.1 Chemical Looping Reactor

The material considered is a nickel catalyst deposited on barium hydride, with the latter component serving as the nitrogen carrier. Ammonia is formed in consecutive steps, as shown by reaction R2. Barium hydride reacts irreversibly with nitrogen gas to form barium imide and hydrogen gas. Following this, the imide is reversibly hydrogenated using hydrogen gas to form ammonia and barium hydride. The material is active at temperatures greater than 250 °C and 1 bar;^22,33^ therefore, the minimum feed temperature is specified to be 250 °C. Barium hydride decomposes into barium metal at temperatures above 600 °C.^44,45^ Its metallic form is not known to be active; therefore, its decomposition is assumed to be undesirable. Thus, the maximum operating temperature is restricted to 600 °C. Heat released from reaction R2a limits the per pass conversion of the nitrogenation reaction, whereas the hydrogenation reaction is limited by its chemical equilibrium.

Chemical looping reactors can be operated in cycles by scheduling multiple reactors,^46,47^ or a continuous output can be obtained with a circulating fluidized bed reactor.^48,49^ The reactor configuration has an impact on the capital costs and energy requirements of the system and is discussed in section 3.3. The energy balance of the reactor is obtained by specifying the outlet temperature and pressure of the reactor. Further details on the reactor model and energy balance calculations are provided in section 2 of the Supporting Information. The outlets of the reactor are specified such that the per pass conversions of both reactions are maximized.

#### 2.2.2 Nitrogenation Loop Separation

With this class of nitrogen-carrying material, hydrogen is generated during the nitrogenation reaction. Hydrogen produced from the reaction should be recycled to the hydrogenation loop because it is of high value and would otherwise accumulate in the nitrogenation loop. Mixtures of hydrogen and nitrogen (3:1 ratio) show reduced rates of ammonia synthesis;^22^ hence, it is assumed that a nitrogen-free hydrogen stream is desirable. The H_2_/N_2_ mixture can be separated via adsorption, distillation, or membranes, which is similar to purge gas recovery processes found in ammonia synthesis units.^41,50^ Membranes have been shown to be cost-effective methods for hydrogen recovery from non-condensable mixtures of gases.^51−53^ Polymer and palladium membranes can be used to selectively separate hydrogen, with the former operating at lower temperatures and the latter operating at temperatures ranging from 300 to 600 °C.^41,52^ The high temperature of the stream is suitable for palladium membranes and would circumvent the need for nitrogen feed preheating. In addition, palladium membranes are highly selective toward hydrogen and can produce a nitrogen-free stream with a purity greater than 99.999%.^54^ The separator is simulated such that a 95% recovery is achieved,^55^ and the outlet pressure of the hydrogen product is equal to its partial pressure at the inlet.

### 2.3 Performance and Cost Evaluation

**Figure 3:**
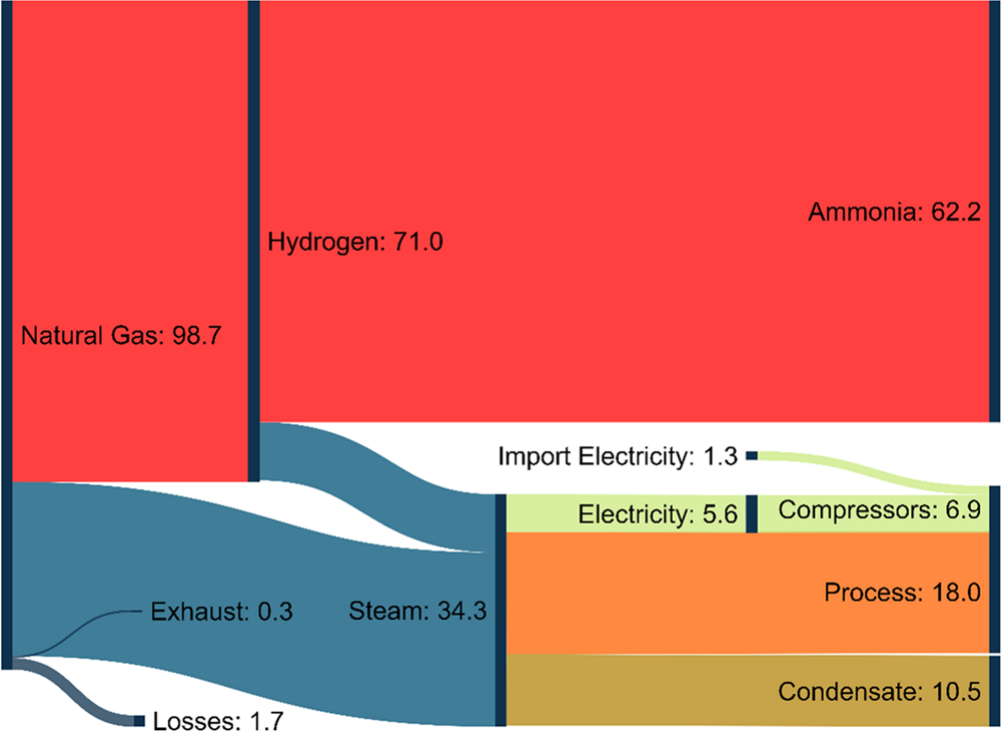
Simplified Sankey diagram showing percentage energy consumptions of the total inputs of the process within a conventional ammonia plant.^56^ Natural gas and imported electricity are the main inputs, with natural gas being the bulk of the energy consumption of the plant.

**Figure 4:**
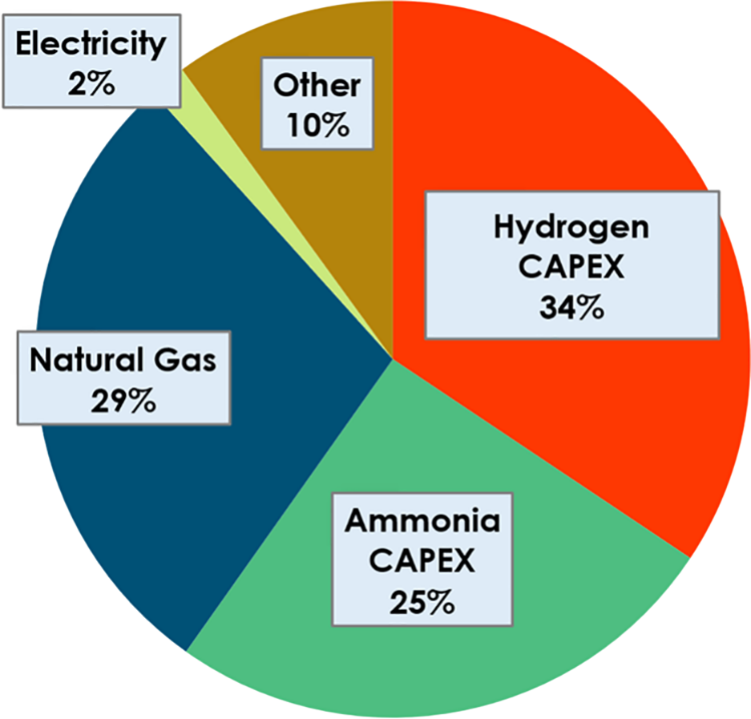
Ammonia product cost breakdown. Cost of production and its constituents were obtained from Pereira et al.^56^ The costing methodology of the United States Department of Energy (DOE) was used to estimate the cost of production based on the capital costs and operating costs of the plant. A capital charge factor of 18.5% was used to obtain these values.

The energy consumption of an ammonia synthesis unit is a key parameter used to benchmark its performance, and it is strongly correlated to its costs because both are predominated by the compressors in the system.^3^ Natural gas is the main energy input (29.1 GJ ton^–1^ of NH_3_), and a small fraction of its energy alongside some imported electricity (0.4 GJ ton^–1^ of NH_3_) is used to drive the compressors (Figure 3). The imported electricity has a negligible impact on the product price compared to other operating costs (e.g., natural gas) (Figure 4). On the other hand, the ammonia synthesis unit constitutes a significant fraction of the capital costs of the plant.^56^ Therefore, the capital cost of the unit dominates the ammonia price, and for this reason, it will be used as a benchmark.

#### 2.3.1 Energy Performance

In the case of the ammonia units, electricity is consumed, while steam is generated. Therefore, the power consumptions of the reference and chemical looping cases are used to evaluate their performances. Mainly, the feed, recycle, and refrigerant compressors are power consumers, whereas steam generated from the reaction is a power generator. The list of assumptions used to calculate the power consumption of the cases is given in section 3 of the Supporting Information (i.e., isentropic efficiencies, mechanical efficiencies, coefficient of performances, and steam pressure levels).
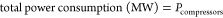
1
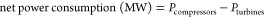
2

#### 2.3.2 Capital Cost Estimation

Equipment costs are calculated using scaling correlations (eq 3)), and the bare module costs of the equipment are calculated using the methodology shown in Table 2. The costing methodology has an accuracy of ±30%, which is typical for parametric cost estimation models.^57^ A list of equations and parameters used for equipment costing is provided in section 5 of the Supporting Information. Costs are updated to 2020 using the Chemical Engineering Plant Cost Index (CEPCI).

**Table 2:** Capital Costing Methodology from Woods’ Rule of Thumb^58^

Equipment costs	
free on-board cost (FOB)	see eq 3
labor and maintenance factor (LM*)	values obtained from Woods^58^
labor and maintenance cost (LM)	LM = FOB × LM*
physical module cost (PM)	PM = LM + 20% FOB
bare module cost (BM)	BM = PM + 20% LM

The chemical looping reactor costs are determined from the number of reactors and the volume of a single fixed bed reactor. The reactor size and quantity are calculated from the reaction rates and their ratios (see section 6 of the Supporting Information). Reaction rate data are limited and extrapolated from Gao et al.^22^ If costs were from a circulating fluidized bed, the reactor costs are higher, which would lead to stricter benchmarks for the chemical looping process. The more conservative cost was chosen to more clearly define material pairings that do not meet the benchmark, which will be discussed further in section 3.2. For the reference case, the reactor size was obtained from Liu.^3^
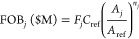
3

### 2.4 Sensitivity Analysis

As a result of their impact on the reaction conversions and the overall energy and economic costs of the system, the sensitivity of the chemical looping process toward the gas–solid reaction thermodynamics was evaluated. Thermodynamic properties, such as the reaction enthalpy and entropy, influence the chemical equilibrium of reactions R2a and R2b. The values of these properties vary with the composition of the material and are partially available for metal hydrides and imides, including BaH_2_/BaNH. The chemical equilibrium is described by the equilibrium temperature, pressure, and yield of the reactions. Hence, these parameters can be used as a proxy to represent the enthalpy and entropy of gas–solid reactions.

The nitrogenation reaction is not limited by equilibrium (see section 9 of the Supporting Information) but instead by the maximum operating temperature of the reactors. The sensitivity toward this factor is beyond the scope of this work and was thus omitted from the analysis. Among the three parameters, only the reaction pressure and ammonia yield of reaction R2b impact auxiliary systems, such as the feed compressors, recycle compressors, and separators, which are the main energy and economic costs within the system. The focus of the study is therefore placed on these two parameters.

### 2.5 Material Property Benchmark

Criteria for the reaction conditions and ammonia yields required to be competitive with the reference case were obtained from the sensitivity study. The required gas–solid thermodynamic properties were calculated from the criteria to benchmark materials with the same reaction scheme and gas phase reaction stoichiometry as reaction R2. Specifically, the required enthalpy (Δ*H*_solid_^°^) and entropy (Δ*S*_solid_^°^) change of the solid species for reaction R2b will be benchmarked. Here, Δ*H*_solid_^°^ is defined as the difference between the formation enthalpies (Δ_f_*H*_*i*_^°^) of the solid products and reactants at the standard state (eq 5). Similar definitions are applied to Δ*S*_solid_^°^, as shown by eq 8.
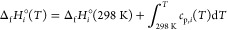
4
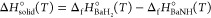
5
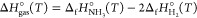
6
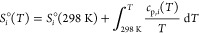
7
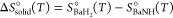
8
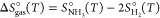
9At the specified temperature, the enthalpy of reaction R2b (Δ_r_*H*°) is composed of Δ*H*_solid_^°^ and the enthalpy change of the gas species in reaction R2b (Δ*H*_gas_^°^) (eq 10). Likewise, this can be applied to the entropy and Gibbs free energy change of reaction R2b (eqs 11 and 12). The Gibbs free energy change of the solid (Δ*G*_solid_^°^) and gas (Δ*G*_gas_^°^) species in reaction R2b can be expressed using eqs 13 and 14.
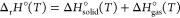
10
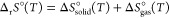
11
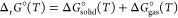
12
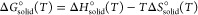
13
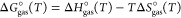
14Substituting eq 13 into eq 12 gives a linear relation between Δ*H*_solid_^°^(*T*) and Δ*S*_solid_^°^(*T*) (eq 15). The value of Δ_r_*G*° is obtained from the required equilibrium ammonia yield of reaction R2b (*Y*), whereas Δ*G*_gas_^°^ is calculated from the National Institute of Standards and Technology (NIST) database.^59^ The estimated changes in Δ*H*_solid_^°^ and Δ*S*_solid_^°^ can be marked on the graph to determine whether the compound can achieve the benchmark.
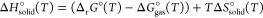
15The required Δ_r_*G*° to achieve the specified value of *Y* can be determined by equating eq 16 to zero at any given reaction temperature (*T*) and pressure (*P*). Solids are assumed to be pure substances; therefore, their activities are taken to be 1. The equilibrium ammonia concentration is calculated from *Y*, as shown in eq 17, and is used to calculate the equilibrium constant associated with reaction R2b (*K*_eq_) using eq 18. Fugacity coefficients (ϕ_*i*_) of the gas species are calculated using the Soave–Redlich–Kwong equation of state.
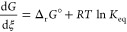
16
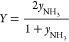
17
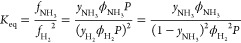
18Materials were identified from the Materials Project database, which could follow the scheme of reaction R2.^60^ This includes the following hydride/imide pairs: LiH/Li_2_NH, MgH_2_/MgNH, CaH_2_/CaNH, SrH_2_/SrNH, and BaH_2_/BaNH. The database was used to obtain the standard enthalpies of formation (Δ_f_*H*_*i*_^°^) and densities of solids (ρ_s_). Standard entropies of solids (*S*_*i*_^°^) were estimated from ρ_s_ using a volume-based thermodynamic method, as shown by eq 19.^61^ The formula unit volume (*V*_m_) (eq 20) is used to estimate the solid entropy. The formation enthalpies and entropies of the solid compounds are estimated at standard temperatures and pressures and used to calculate Δ*H*_solid_^°^ and Δ*S*_solid_^°^.
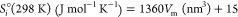
19
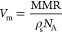
20

## 3 Results and Discussion

### 3.1 Power Consumption

Power consumers and generators for the reference case (ref case) and chemical looping case (CL case) are shown in Table 3. The CL case(H_2_) and CL case(N_2_) correspond to the hydrogenation and nitrogenation loops, respectively. The total and net power consumptions of the CL case are 18 and 11% higher than the ref case, respectively. The difference between the ref case and CL case is mainly due to the increased feed and recycle compression duties. Furthermore, the hydrogenation loop accounts for 85% of the total power consumption of the unit, and this loop alone has a similar power consumption to the reference case.

**Table 3:** Performance Comparison between the Reference Ammonia Synthesis Unit (Ref Case) and the Chemical Looping Ammonia Synthesis Unit (CL Case)^a^

plant performance results	ref case	CL case	CL case(H_2_)	CL case(N_2_)
reactor conversion (%)	26.2	-	26.1	15.7
feed compressors (MW)	12.0	14.9	12.0	2.9
recycle compressors (MW)	1.9	3.2	2.1	1.1
refrigeration compressor (MW)	8.6	8.4	8.4	
steam turbines (MW)	–8.0	–10.4		–10.4
total power consumption (MW)	22.5	26.5	22.5	4.0
net power consumption (MW)	14.5	16.1		

a The energy consumptions and reaction conversions per pass associated with the hydrogenation loop [(CL case(H_2_)] and nitrogenation loop [CL case(N_2_)] are shown.

In comparison to ref case, the hydrogen conversion per pass is equal, while the nitrogen conversion per pass is lower because it is limited by the maximum operating temperature. Consequently, more energy is required for nitrogen recompression and recycling hydrogen produced from the membrane separator to the hydrogenation loop. A 4.0 MW increase in power consumption is obtained with the CL case compared to the ref case. The nitrogenation reaction is non-equilibrium-limited (see section 9 of the Supporting Information), and if a 100% conversion per pass was assumed to be possible, the power consumption of the CL case would reduce by 4.3 MW. Although unlikely, this would only improve the power consumption of the CL case such that it is equal to the ref case.

The total power consumptions are underestimated by 30% compared to Nielsen^6^ and DOE.^40^ This could be due to uncertainties associated with the omission of inert compounds, compressor efficiencies, and the coefficient of performance of the refrigeration unit. However, the error associated with these assumptions should impact both the ref case and CL case similarly; therefore, the difference between the two would not change significantly.

These comparisons suggest that either a near-complete nitrogen conversion per pass is required to reach an equivalent power consumption to the ref case or a higher hydrogen conversion per pass is key to obtaining a similar or improved power consumption to the ref case. As a result of their identical configurations and distribution of power consumptions, it can be surmised that the performance of the hydrogenation loop can be improved in the same manner as the conventional process, i.e., by increasing the ammonia yield.

### 3.2 Capital Cost

A summary of equipment costs is given in Table 4, and a detailed list is given in section 5 of the Supporting Information. Initial comparisons suggest that the CL case has a marginally lower (4%) capital cost than the ref case. The largest costs in both the ref case and CL case were from the compressors and ammonia separator; note that the latter includes a compressor. Capital costs of compressors were 22% higher for the CL case and are due to the lower conversion per pass of reaction R2a and higher feed compressor duties. The majority of capital costs in the CL case are in the hydrogenation loop, which constitutes 79% of the capital costs. Hence, from both an energy and economic perspective, there is incentive to reduce compression duties and more so with the CL case. In addition, reducing the energy consumptions in the hydrogenation loop can achieve the greatest cost savings.

**Table 4:** Capital Cost Comparison between the Reference Ammonia Synthesis Unit (Ref Case) and the Chemical Looping Ammonia Synthesis Unit (CL Case)^a^

capital costs	ref case	CL case	CL case(H_2_)	CL case(N_2_)
compressors ($M)	36.0	43.8	38.8	4.0
reactors ($M)	15.0	4.0^b^		
separators ($M)	22.5	24.0	22.2	1.8
heat exchangers ($M)	10.5	8.6	7.6	1.0
total ($M)	84.0	80.4		

a The capital costs associated with the hydrogenation loop [CL case(H_2_)] and nitrogenation loop [CL case(N_2_)] are shown.

b The value corresponds to multiple fixed bed reactors. With circulating fluidized bed reactors, it is estimated to be 14.8 $M.

Even though the uncertainties associated with the cost methodology can be up to 30%, the relative deviation of the ref case and CL case from the true values should be similar because most unit operations involved are comparable. However, the reactor configuration and cost correlation are different, which can introduce error in the difference observed between the ref case and CL case. The correlation used to estimate reactor costs in ref case accounts for the complexities in its design, such as the radial flow, multi-bed support, and interbed cooling. A multi-bed system with intercooling is necessary to achieve high yields; otherwise, the exothermic nature of the reaction lowers the equilibrium ammonia yield of the reaction, whereas in the CL case, the nitrogenation reaction is exothermic, non-equilibrium-limited, and separate from the ammonia synthesis step; hence, no intercooling is required.

The uncertainties associated with the reaction rates used for the reactor sizing are large because it was extrapolated from lab-scale experiments performed at 1 bar, 200–350 °C, and high space velocities.^22^ Although high-pressure operations can increase the rate of the hydrogenation reaction,^33^ the apparent reaction rate will be reduced by resistances as a result of mass transport phenomena, operation close to chemical equilibria, and degradation of hydrides and imides (e.g., irreversible oxidation by oxygenated species). On balance, the reaction rates used here are likely optimistic and, therefore, lead to a conservative estimate of the reactor size. While further work is required to accurately establish the reactor size and costs, for the purpose of the material property benchmarking, a conservative estimate will rule out fewer material pairings, which may be suitable for this process. This is appropriate given that there are large uncertainties in the estimated thermodynamic properties of these materials, as will be discussed in section 3.5.

### 3.3 Impact of Reactor Configuration

Presently, the reactor model closely resembles a circulating fluidized bed reactor because steady-state unit operations and uniform solid temperatures are assumed. If the reactor was modeled with multiple fixed bed reactors, the cyclic configuration would lower the outlet temperature of reaction R2a and increase it for reaction R2b. A cyclic configuration was chosen for the reactor size and cost because circulating fluidized bed reactors face issues operating at high pressures.^56^ Hence, the impact of the outlet temperature of the chemical looping reactor was considered, and it was found to have no effect on the total power consumption of the CL case because the compressor operating conditions are isolated from the reactor. However, it can impact the power output of the turbine by decreasing the maximum temperature of the superheated steam. In addition, the non-uniform temperature of the reactor would affect the maximum equilibrium yield of reaction R2b and will be considered in the material property benchmarking. From a capital cost point of view, the cost with a circulating fluidized bed reactor was found to be higher than with multiple fixed bed reactors (14.8 versus 4.0 $M).

### 3.4 Sensitivity Analysis

The impact of the chemical looping reaction thermodynamics on the total power consumption and capital costs is described hereon. Because compressors were found to be the main contributors to the power consumption and capital costs of the system, the operating temperatures of the reactors were omitted from this analysis and only variations with ammonia yield and synthesis loop pressure were observed.

#### 3.4.1 Power Consumption Sensitivity

**Figure 5:**
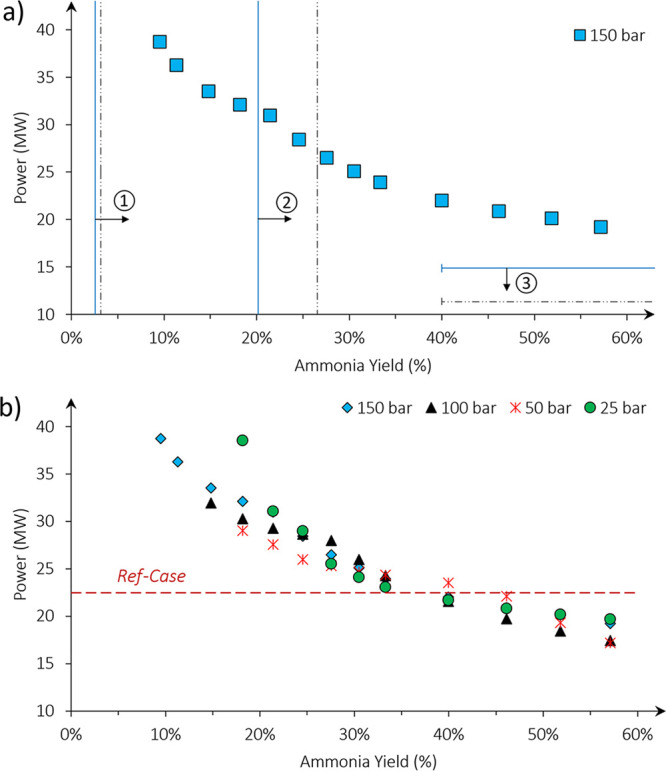
(a) Variations in total power consumption with the ammonia yield for the CL case at 150 bar. The lines describing the trend are determined by ① the ammonia vapor pressure at −30 °C, ② the ammonia vapor pressure at 30 °C, and ③ the minimum power consumption at an ammonia yield of 100%. The arrows indicate how these lines shift with decreasing pressure, and the dotted lines correspond to lines for the CL case at 100 bar. (b) Sensitivity of the total power consumption of chemical looping processes toward the synthesis loop pressure and ammonia yield. The results are compared to the power consumption of the initial ref case.

Although the CL case was less favorable in terms of total power consumption and capital costs, the previous comparison suggests that higher ammonia yields are key to achieving a more competitive process. The variation in power consumption with the ammonia yield for CL case is shown in Figure 5a. The trend in power consumption for a given pressure can be described by three defining features and is equal in both the ref case and CL case. The beginning of the trend is marked by a vertical asymptote, which represents the vapor pressure of ammonia in the refrigeration unit (−30 °C). At sufficiently high ammonia vapor pressures, the ammonia gas is partially condensable by cooling water (30 °C); hence, at 18% yield and 150 bar, there is a sudden decrease in power consumption. At a 100% yield, a horizontal asymptote is formed, which equals the power consumption of feed compressors and recycle compressors in the nitrogenation loop. Decreases in synthesis loop pressures cause the vapor pressures of ammonia to increase and feed compressor duties to decrease (see section 7 of the Supporting Information). This causes a positive horizontal shift in the trend and some stretching because the change in vapor pressure is not linear with pressure.

In comparison to the ref case (Figure 5b), yields of at least 36% are required to achieve a power consumption similar to the CL case. In the conventional reaction, this would require catalysts with better kinetics at lower temperatures, whereas in the chemical looping process, it depends upon both the reaction thermodynamics and kinetics of reaction R2b. When variations in the synthesis loop pressure are considered (Figure 5b), it is found that power consumptions at a given yield do not vary as significantly with pressure and an optimal synthesis loop pressure exists at a given yield. This invariance is due to the trade-off between the energy required for feed compression and that required for separation and recycling. To synthesize ammonia at low pressures and temperatures is a widely promoted goal in the literature; however, the results indicate that the maximum ammonia yield achievable is a key parameter when developing new materials and processes for ammonia synthesis. This constraint has not been generally recognized until now.

#### 3.4.2 Capital Cost Sensitivity

**Figure 6:**
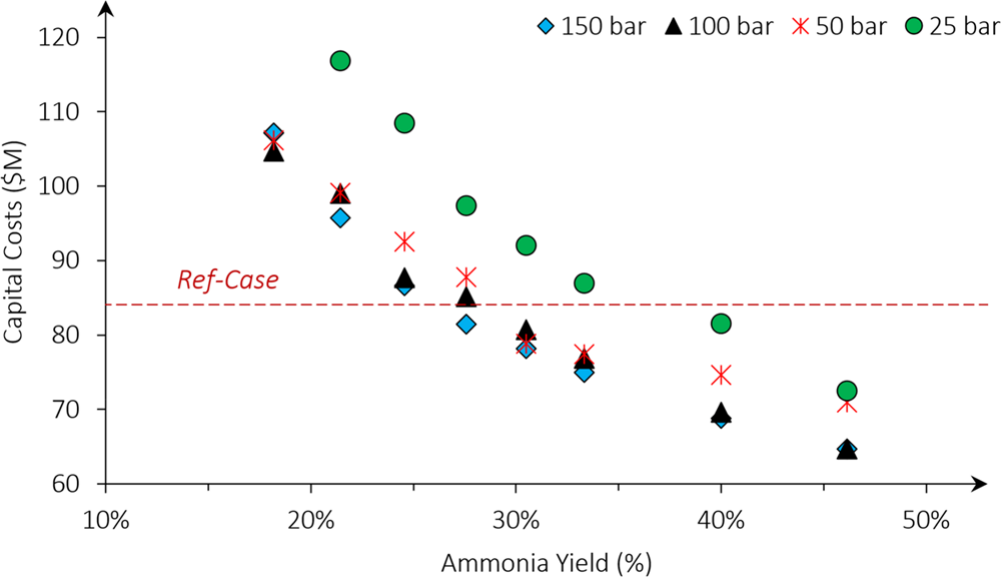
Sensitivity of the capital costs of chemical looping processes toward the synthesis loop pressure and ammonia yield. The results are compared to the power consumption of the ref cases.

The capital cost variation with the yield for the initial CL case (Figure 6) appears to be correlated with the power consumption of the unit. Although the relative decreases in both benchmarks are similar, the capital costs predominate the ammonia price and are therefore more significant. As the synthesis loop pressure decreases, the results remain invariant at 50–150 bar and 18–33% yield. Similarly, this invariability is due to the trade-off between the feed compression duty and the separation and recycling duties.

A significant difference in capital costs is observed at 25 bar, which decreases with the ammonia yield. The increase is due to larger hydrogen recycling duties and lower outlet temperatures for the hydrogenation reaction, which increased capital costs of the compressors and hydrogen preheater, respectively. The latter is a result of the given design and could be reduced by preheating the feed of the hydrogenation reactor with heat from the nitrogenation loop. However, the increase in recycle compression costs are due to the ammonia yield and synthesis loop pressure. To operate at lower pressures, higher yields are required to maintain low capital costs.

The invariability of the capital costs with pressure indicates that the yield is a better indicator of capital costs. The capital costs are thus more sensitive to ammonia yields and would be a more useful criteria for benchmarking material properties. In comparison to the ref case whose yield is 26%, an equivalent capital cost is achieved at ammonia yields of >26% with the CL case, and this value will therefore be used to benchmark the required gas–solid reaction thermodynamics.

### 3.5 Material Property Benchmark

**Figure 7:**
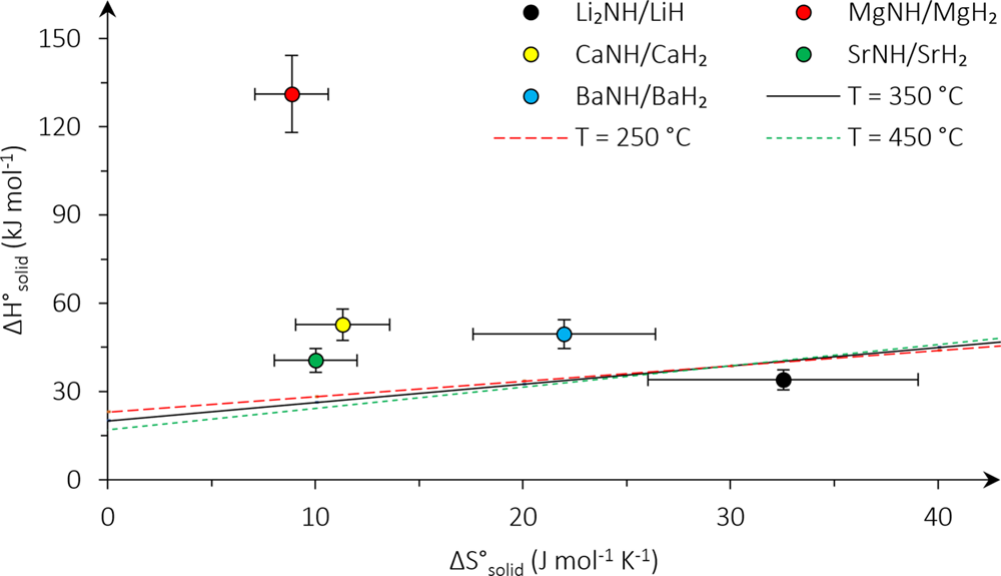
Minimum Δ*H*_solid_^°^ and Δ*S*_solid_^°^ are represented by the plotted lines. Pairings situated below or to the right of these lines have properties enabling them to have comparable or better performance than the conventional process. Three temperatures are considered for the benchmark at 150 bar.

The plotted lines in Figure 7 represent Δ*H*_solid_^°^ and Δ*S*_solid_^°^ required to achieve a greater than 26% ammonia yield at a given temperature and 150 bar. Materials with Δ*H*_solid_^°^ and Δ*S*_solid_^°^ values situated below the lines drawn in Figure 7 have potentially greater ammonia yields than the conventional reaction. The chosen temperatures are based on the simulation of the CL case if circulating fluidized beds are considered (*T* = 350 °C), if fixed beds are considered (*T* = 450 °C), and if a milder temperature is feasible (*T* = 250 °C). Only the LiH/Li_2_NH pair satisfies these conditions, whereas other pairings do not meet these criteria. The present estimates for LiH/Li_2_NH give a yield of 62% at 350 °C and 150 bar or an equal yield to ref case at 50 bar. On the basis of the sensitivity analysis, the power consumption and capital costs may be reduced 14 and 21%, respectively. The exact values of these properties under relevant conditions would need to be determined experimentally to affirm these results.

Errors associated with the estimation method were determined by comparing the calculated values to values in the literature (section 8 of the Supporting Information). An average uncertainty of 10 and 20% was found for Δ*H*_solid_^°^ and Δ*S*_solid_^°^, respectively. Despite this uncertainty, pairs such as MgH_2_/MgNH, CaH_2_/CaNH, SrH_2_/SrNH, and BaH_2_/BaNH fail to meet the benchmark. Only the LiH/Li_2_NH pair is the most likely candidate to meet this benchmark. It should be noted that solid properties presented in Figure 7 were estimated at standard conditions (25 °C and 1 bar), which may have systematic errors as a result of the contribution of heat capacities. The Dulong–Petit law can be used to estimate the heat capacity of the solids at higher temperatures.^62,63^ Note that the hydrides and imides have an equal number of atoms, which would yield a zero value for the error. Instead, if the contribution of the hydrogen atom to the solid heat capacity was excluded (because its contribution is assumed the smallest), errors of −8.2 kJ mol^–1^ and −18.8 J mol^–1^ K^–1^ for Δ*H*_solid_^°^ and Δ*S*_solid_^°^, respectively, can be expected. This corresponds to an error of +3.5 kJ mol^–1^ for Δ*G*_solid_^°^ at a temperature of 350 °C and a yield of 51% for LiH/Li_2_NH. Note that these systematic errors are not displayed on Figure 7.

Overall, alkali and alkaline earth metal hydride/imide pairings seem unlikely to provide better performances based on the analysis herein. A CLAS process could be viable if nitrogen carriers with more favorable thermodynamic properties were used. If Δ*H*_solid_^°^ and Δ*S*_solid_^°^ favored the hydrogenation reaction (reaction R2b), it would reduce the equilibrium conversion of the nitrogenation reaction (reaction R2a). However, because reaction R2a is not limited by equilibrium (section 9 of the Supporting Information), material pairings that favor the equilibrium of reaction R2b can be considered. Alternatively, nitrogen carriers using transition metals could provide better yields if the required Δ*H*_solid_^°^ and Δ*S*_solid_^°^ are present.

## 4 Conclusion

With chemical looping ammonia synthesis, the introduction of a solid intermediate alters the equilibrium conversions of the reaction and affects the performance of the process. The impact of the solid properties on the energy consumption and capital costs of the CLAS process was evaluated using the equilibrium conditions (yield, pressure, and temperature) of reaction R2b as a proxy. The ammonia yield was found to be the key factor affecting the ammonia synthesis process, and a greater than 26% yield is required to compete with the reference case. This criterion is a conservative value because reactor costs are likely underestimated for the chemical looping case; however, this approach more clearly defines material pairings whose solid properties are unsuitable for the process. Through this methodology, LiH/Li_2_NH has been identified as a potentially viable nitrogen-carrying material for chemical looping ammonia synthesis, although experimental work is required to validate whether equilibrium ammonia yields of 62% are achievable. In addition, this paper serves to highlight the need to perform appropriate evaluation of candidate chemical looping ammonia synthesis approaches. Kinetic work alone, regardless of how promising, at best is unlikely to prove useful and at worst can be misleading.

## Supporting Information Available

Comparison of reaction rates of the ammonia synthesis catalyst (section 1), chemical looping ammonia synthesis reactor energy balance and implementation in Aspen Plus (section 2), simulation parameters and assumptions (section 3), process flow diagrams and mass balances (section 4), equipment cost correlations and detailed equipment costs (section 5), chemical looping reactor sizing (section 6), minimum yield required to condense ammonia gas at a given synthesis loop pressure (section 7), material property estimation uncertainty (section 8), and material property benchmarking for the nitrogenation reaction (section 9) (PDF)
