# Supplementary material for: Impact of Gas–Solid Reaction Thermodynamics on the Performance of a Chemical Looping Ammonia Synthesis Process
Source: Energy Fuels. 2022 Jul 1;36(17):9757–67. doi: 10.1021/acs.energyfuels.2c01372 (PMC9442650; doi:10.1021/acs.energyfuels.2c01372)
Supplement: Supplementary file 1 — ef2c01372_si_001.pdf [file ef2c01372_si_001.pdf]

Impact of gas-solid reaction thermodynamics on performance of a chemical looping ammonia synthesis process

Reinaldo Juan Lee Pereira, Wenting Hu, and Ian S. Metcalfe

School of Engineering, Newcastle University, Newcastle Upon Tyne NE17RU, United Kingdom

Supplementary Information Contents:

1. Comparison of ammonia synthesis catalysts S2
2. Chemical looping ammonia synthesis reactor energy balance S3
   2.1 Conventional reaction's energy balance S3
   2.2 Chemical looping ammonia synthesis reaction's energy balance S4
   2.3 Aspen Plus Implementation S6
3. Simulation parameters S7
4. Process Flow Diagram & Mass Balances S8
   4.1 Reference case flow diagram & mass balance: S8
   4.2 Chemical looping case flow diagram & mass balance: S9
5. Cost Correlations and detailed equipment costs: S10
6. Chemical looping reactor sizing S11
7. Vapour-liquid equilibrium of ammonia in hydrogen S12
8. Material property uncertainty: S13
9. Nitrogenation reaction material benchmark: S14

Supplemental references S15

1. Comparison of ammonia synthesis catalysts

Table S1 Summary of ammonia synthesis performance of novel catalysts.

Catalysts	r_{NH3} (mmol. $g^{-1}.h^{-1}$)	NH_3 yield (%)	Reaction Conditions	WHSV (ml. $g^{-1}.h^{-1}$)	Reference
**Chemical Looping**					
Pd-Li_2NH	6.9	0.28	300°C, 1 bar	60000	(1)
Mn_4N-BaH_2	13.1	0.53	300°C, 1 bar	60000	(2)
Mn_4N-LiH	23.7	0.97	300°C, 10 bar	60000	(2)
Mn_4N-LiH	15.8	0.64	300°C, 1 bar	60000	(2)
BaH_2-Ni/Al_2O_3	17.6	0.71	300°C, 1 bar	60000	(3)
BaH_2-Ni	9.4	0.38	300°C, 1 bar	60000	(3)
Ni-LiH	4.6	0.19	300°C, 1 bar	60000	(3)
Ni_3ZnN	2.9	0.39	400°, 1 bar	18000	(4)
**Conventional**					
Ru(10%)/Ba-Ca$(NH_2)_2$	23.3	3.16	300°C, 9 bar	36000	(5)
Co(8%)/Ba-Ca$(NH_2)_2$	6.6	0.90	300°C, 9 bar	36000	(5)
Ru(2%)/C12A7:e^-	0.76	0.10	300°C, 9 bar	36000	(5)
Fe-LiH	5.0	0.41	300°C, 10 bar	60000	(6)
Co-LiH	4.7	0.38	300°C, 10 bar	60000	(6)
Ni-LiH	0.1<	0.08<	300°C, 10 bar	60000	(6)
BaH_2-Co(5%)/CNTs	4.8	0.39	300°C, 10 bar	60000	(7)
Li-Mn-N	0.87	0.07	400°, 1 bar	12000	(8)
Co_3Mo_3N-Cs	4.8	0.39	300 °C, 10 bar	9000	(9)
Co_3Mo_3N	2.4	0.20	300 °C, 10 bar	9000	(9)
Fe-K_2O-Al_2O_3	2.0	0.16	300 °C, 10 bar	9000	(9)
Industrial Fe Catalyst	5.4	0.74	300°C, 9 bar	36000	(5)
Ru(10%)-Cs/MgO	0.6	0.08	300°C, 9 bar	36000	(5)

2. Chemical looping ammonia synthesis reactor energy balance

2.1 Conventional reaction's energy balance

The conventional reaction's (R1) outlet conditions (Temperature and Pressure) can be determined by considering the enthalpy changes required to go from states 1 to 2, 2 to 3, and 3 to 4 (Figure S1). The change in enthalpy from states 1 to 2 corresponds to the change in temperature and pressure of the reactants from the inlet conditions to standard conditions. The change in enthalpy from 2 to 3 corresponds to the enthalpy change of reaction R1 under standard conditions. The change in enthalpy from 3 to 4 corresponds to change in temperature and pressure of the products from standard conditions to outlet conditions. The sum of these 3 enthalpy changes should correspond to the change from states 1 to 4 which is equal to 0 for an adiabatic process.

$$3H_{2(g)} + N_{2(g)} \rightleftharpoons 2NH_{3(g)} \quad \Delta_r H^{\circ} = -46.0\ kJ\ mol^{-1} NH_3 \qquad (R1)$$

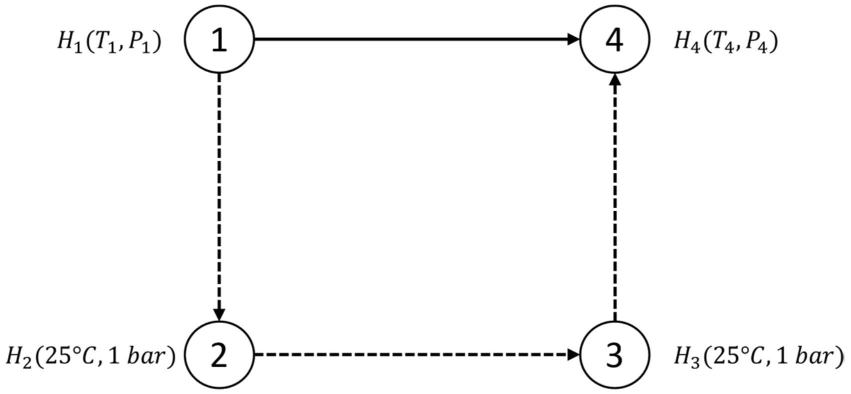

Figure S1 Changes in state of the conventional ammonia synthesis reaction assuming adiabatic conditions.

Table S2 Nomenclature for the energy balance of the conventional ammonia synthesis reaction's energy balance. A similar nomenclature is used for the chemical looping reactions energy balance.

**Definition**	**Nomenclature**
Enthalpy of reactants at feed conditions (State 1)	H_1
Enthalpy of reactants under STP (State 2)	H_2
Enthalpy of products under STP (State 3)	H_3
Enthalpy of products at outlet conditions (State 4)	H_4
Enthalpy Change from state 1 to 4	$\Delta H_{14} = H_4 - H_1 = 0$
Enthalpy Change from state 1 to 2	$\Delta H_{12} = \mathrm{H}_2 - \mathrm{H}_1 = \sum_i n_i \int_{T_1}^{T_2} c_p(T)dT$
Enthalpy Change from state 2 to 3	$\Delta H_{23} = \mathrm{H}_3 - \mathrm{H}_2 = \Delta_r H(T_2, P_2) = \Delta_r \mathrm{H}(T_3, P_3)$
Enthalpy Change from state 3 to 4	$\Delta H_{34} = \mathrm{H}_4 - \mathrm{H}_3 = \sum_i n_i \int_{T_3}^{T_4} c_p(T)dT$

2.2 Chemical looping ammonia synthesis reaction's energy balance

Unlike the conventional reaction, the energy balance of the chemical looping reaction is split into 2 reaction steps. The outputs of the reactions are coupled and must be solved together to obtain a solution for the outlet conditions (States 4, Figure S2). The unknowns are the outlet temperatures of gaseous products from R2a and R2b and their solid inlet/outlet temperatures. If it is assumed that the gas-solid are thermally equilibrated and that the solid temperature is uniform throughout the reactor bed, the outlet temperature of the gas and solid for either R2a or R2b are equal. Additionally, the inlet temperature of the solid for R2a is equal to the outlet temperature of the solid or gas for R2b.

$$BaH_{2(s)} + \frac{1}{2}N_{2(g)} \rightarrow BaNH_{(s)} + \frac{1}{2}H_{2(g)} \quad \Delta_r H^{\circ} = -46.3\ kJ\ mol^{-1}\ NH_3 \qquad (R2a)$$

$$BaNH_{(s)} + 2H_{2(g)} \rightleftharpoons BaH_{2(s)} + NH_{3(g)} \quad \Delta_r H^{\circ} = +0.3\ kJ\ mol^{-1}\ NH_3 \qquad (R2b)$$

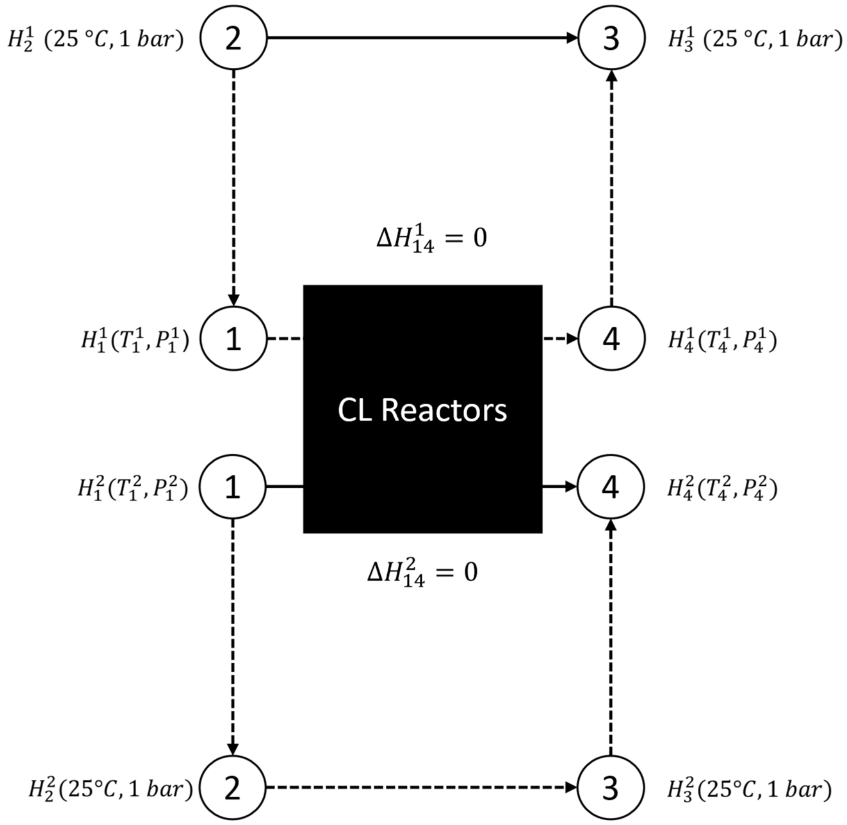

Figure S2 Changes in state for the chemical looping reaction under adiabatic conditions. The superscript value denotes the nitrogenation reaction (1) and hydrogenation reaction (2)

Table S3 Additional nomenclature used for the chemical looping reactions energy balances. Refer to Table 1 for the general nomenclature used. The nomenclature shown here is meant to differentiate between the reaction steps and solid and gas reactants/products.

**Item**	**Nomenclature**	**Definition**
Subscripted numbers	e.g., ΔH_{12}	Steps or states shown in the enthalpy change diagrams.
Superscripted numbers	e.g., ΔH_{12}^1	1 = Nitrogenation R2a<br>2 = Hydrogenation R2b
Subscripted letters	$g, s, and\ i$	Gas species, solid species, and all species, respectively.
Series	g_{in} and g_{out}<br>$s_{in}\ and\ s_{out}$	Gas species at the inlet (States 1,2) and outlet (States 3,4) of the reaction.<br>Solid species at the inlet (States 1,2) and outlet (States 3,4) of the reaction.

For R2a which is marked by a superscripted number of one, the change in enthalpy between the products and reactants (ΔH_{14}^{1}) is equal to 0 since the process is assumed to be adiabatic. Therefore, the unknowns remaining are changes from states 1 to 2, 2 to 3, and 3 to 4. The change in enthalpy is separated into its gaseous and solid phase e.g. $\Delta H_{34}^{1} = \Delta H_{34g}^{1} + \Delta H_{34_s}^{1}$ where ΔH_{34g}^{1} represents the change in enthalpy from state 3 to 4 of the gaseous products and $\Delta H_{34_s}^{1}$ represents the change in enthalpy from state 3 to 4 of the solid products.

$$\Delta H_{14}^{1} = \Delta H_{12}^{1} + \Delta H_{23}^{1} + \Delta H_{34}^{1} = 0 \quad (1)$$

$$\Delta H_{34}^{1} = -(\Delta H_{12}^{1} + \Delta H_{23}^{1}) \quad (2)$$

$$\Delta H_{34g}^{1} + \Delta H_{34_s}^{1} = -\left(\Delta H_{12_g}^{1} + \Delta H_{12_s}^{1} + \Delta H_{23_g}^{1} + \Delta H_{23_s}^{1}\right) \quad (3)$$

Assuming that gases are ideal, the change in enthalpies from states 1 to 2 and from states 3 to 4 for both gases and solids are due to changes in temperature. For the change in enthalpy from state 2 to 3 it is equivalent to the standard enthalpy change of reaction. Substituting these changes in enthalpy with the corresponding equations yields equation (4). In addition, it is also assumed that the heat capacity of the solid is constant with temperature and that the heat capacity of the solid reactants and products are equal (Equation (5) and (6), respectively).

$$\sum_{g_{out}} \int_{T_3}^{T_4^1} n_g^1 c_{p_g}^1(T)dT + \sum_{s_{out}} \int_{T_3}^{T_4^1} n_s^1 c_{p_s}^1 dT = -\left[\sum_{g_{in}} \int_{T_{1_g}^1}^{T_2} n_g^1 c_{p_g}^1(T)dT + \sum_{s_{in}} \int_{T_{1_s}^1}^{T_2} n_s^1 c_{p_s}^1 dT + \xi^1 \Delta_r H^1\right] \quad (4)$$

$$\sum_{g_{out}} \int_{T_3}^{T_4^1} n_g^1 c_{p_g}^1(T)dT + n_s^1 c_{p_s}^{\ 1}(T_4^1 - T_3) + n_s^1 c_{p_s}^1\left(T_2 - T_{1_s}^1\right) = -\sum_{g_{in}} \int_{T_{1_g}^1}^{T_2} n_g^1 c_{p_g}^1(T)dT - \xi^1 \Delta_r H^1 \quad (5)$$

$$\sum_{g_{out}} \int_{T_3}^{T_4^1} n_g^1 c_{p_g}^1(T)dT + n_s^1 c_{p_s}^{\ 1}\left(T_4^1 - T_{1_s}^1\right) = -\sum_{g_{in}} \int_{T_{1_g}^1}^{T_2} n_g^1 c_{p_g}^1(T)dT - \xi^1 \Delta_r H^1 \quad (6)$$

Equations (7) and (8) contain 4 terms which can be determined using appropriate unit operation blocks in Aspen Plus. Note that the term corresponding to the enthalpy change of the solids from state 1 to 4 are of equal value in equations (7) and (8). Heat capacities of gases can be calculated in Aspen plus, however the solid heat capacities are calculated using Dulong-Petit's rule. In addition, the enthalpy of reaction is calculated using values from literature.

$$\sum_{g_{out}} \int_{T_3}^{T_4^1} n_g^1 c_{p_g}^1(T)dT = -\sum_{g_{in}} \int_{T_{1_g}^1}^{T_2} n_g^1 c_{p_g}^1(T)dT - \xi^1 \Delta_r H^1 - n_s^1 c_{p_s}^{\ 1}\left(T_4^1 - T_{1_s}^1\right) \quad (7)$$

$$\sum_{g_{out}} \int_{T_3}^{T_4^2} n_g^2 c_{p_g}^2(T)dT = -\sum_{g_{in}} \int_{T_{1_g}^2}^{T_2} n_g^2 c_{p_g}^2(T)dT - \xi^2 \Delta_r H^2 - n_s^2 c_{p_s}^{\ 2}\left(T_4^2 - T_{1_s}^2\right) \quad (8)$$

2.3 Aspen Plus Implementation

The mathematical description of the energy balance is meant to describe the unit operations required in Aspen Plus. A flowsheet for the chemical looping reactors is given in Figure S3 and the function of each unit operation is shown in Table S4. Unit operations performed in Excel are calculated iteratively within the process simulator. The specified inputs are thus the extent of reaction (ξ), enthalpy of reaction ($\Delta_r H$), and solid heat capacity (c_{p_s}).

Table S4 List of mathematical terms present in equation (2) and their corresponding unit operations in Aspen Plus.

Definition	Symbol or Equation	Unit Operation	Identity
$Enthalpy\ of\ state\ 1$	H_1	Stream	R2-H1
$Enthalpy\ of\ state\ 2$	H_2	Stream	R2-H2
$Enthalpy\ of\ state\ 3$	H_3	Stream	R2-H3
$Enthalpy\ of\ state\ 4$	H_4	Stream	R2-H4
$Enthalpy\ change\ of\ gas\ products\ (3 \rightarrow 4)$:	$\sum_{g_{out}} \int_{T_3}^{T_4^2} n_g^2 c_{p_g}^2(T)dT$	Heat exchanger block	R2-H34
$Enthalpy\ change\ of\ gas\ reactants\ (1 \rightarrow 2)$:	$-\sum_{g_{in}} \int_{T_{1_g}^2}^{T_2} n_g^2 c_{p_g}^2(T)dT$	Heat exchanger block	R2-H12
$Enthalpy\ change\ of\ reaction\ (2 \rightarrow 3)$:	$-\xi^2 \Delta_r H^2$	Excel sheet/Stream	R2-H23
$Enthalpy\ change\ of\ solid\ \ (1 \rightarrow 4)$:	$-n_s^2 {c_{p_s}}^2 (T_4^2 - T_{1_s}^2)$	Excel sheet/Stream	S

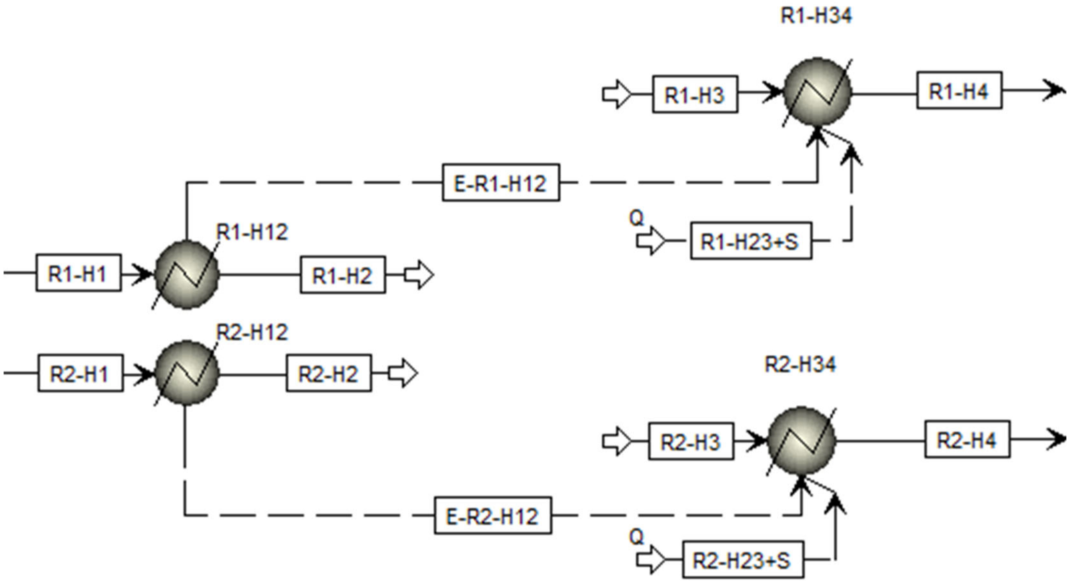

Figure S3 Aspen Plus model of the chemical looping reactions. The top half (R1) corresponds to the nitrogenation reaction whilst the bottom half (R2) corresponds to the hydrogenation reaction.

3. Simulation parameters

The equation of state was chosen based on an Aspen Plus model in literature[10]. The compressor and turbine efficiencies were taken from sources [11,12] and were varied based on the steam turbines outlet conditions. Water used to generate steam is pre-heated in the same heat exchangers as the evaporators since there was no impact on the quantity of steam produced and a negligible impact on the costs of the system. The coefficient of performance for ammonia-based refrigeration is obtained from Ouadha et al. [13] for an evaporator temperature of -30 °C. The solid heat capacity is found using Dulong-Petit's rule for the $BaH_2/BaNH$ pair. A complete solid conversion is assumed in the simulation. A 95% hydrogen recovery was specified based on Emerson et al.[14]. The overall heat transfer coefficients were obtained from Sinnott & Towler[15].

Table S5 List of parameters used when simulating the reference and chemical looping cases.

**Simulation Parameters**	**Input**
Equation of state	RKS-BM
Compressor isentropic efficiency (%)	85
Compressor mechanical efficiency (%)	99
Turbine isentropic efficiency (%)	HP (40 bar) = 80<br>MP (15 bar) = 75<br>LP (6 bar) = 70<br>Cond (0.05 bar) = 60
Turbine mechanical efficiency (%)	99
Coefficient of performance for NH_3 refrigeration	2
Solid heat capacity (J mol^{-1} K^{-1})	74.8
Solid conversion (%)	100
Membrane unit hydrogen recovery (%)	95
Overall heat transfer coefficient (W m^{-2} K^{-1})	Gas – Gas = 300<br>Gas – Liquid = 400<br>Liquid – Liquid = 1000

4. Process Flow Diagram & Mass Balances

4.1 Reference case flow diagram & mass balance:

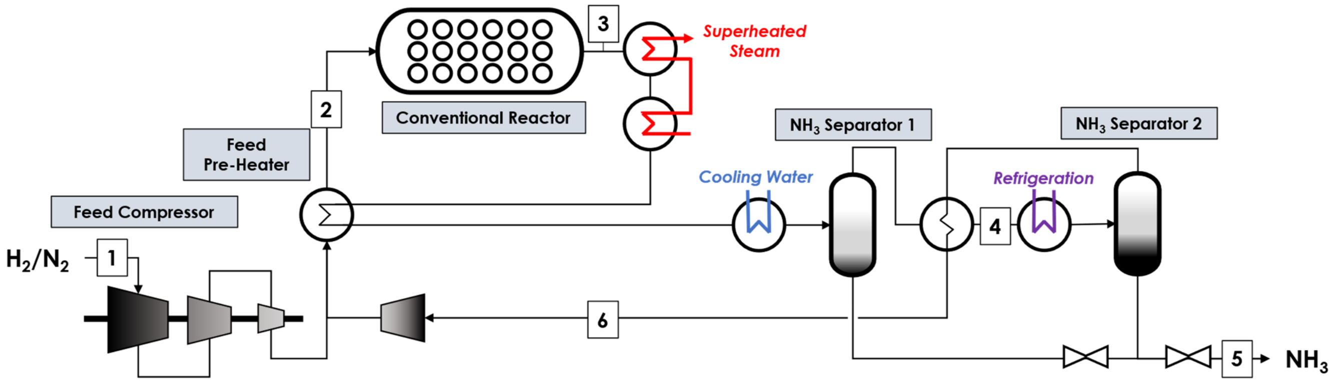

Figure S4 Process flow diagram of the reference ammonia synthesis unit.

Table S6 Mass balance of the reference ammonia synthesis unit.

#ID	P	T	N	m	MW	Composition (% mol)		
	**bar**	**°C**	**kmol/s**	**kg/s**	**kg/kmol**	**NH_3**	**N_2**	**H_2**
1	25.0	25.0	2.00	17.0	8.52		25.0	75.0
2	149.7	338.3	7.62	65.6	8.61	1.1	24.7	74.2
3	145.2	510.0	6.62	65.6	9.90	16.3	20.9	62.8
4	139.5	13.3	6.32	60.5	9.57	12.3	21.9	
5	200.0	-8.6	0.99	16.9	17.02	99.9		0.01
6	135.1	24.5	5.62	48.6	8.64	1.5	4.6	73.9

4.2 Chemical looping case flow diagram & mass balance:

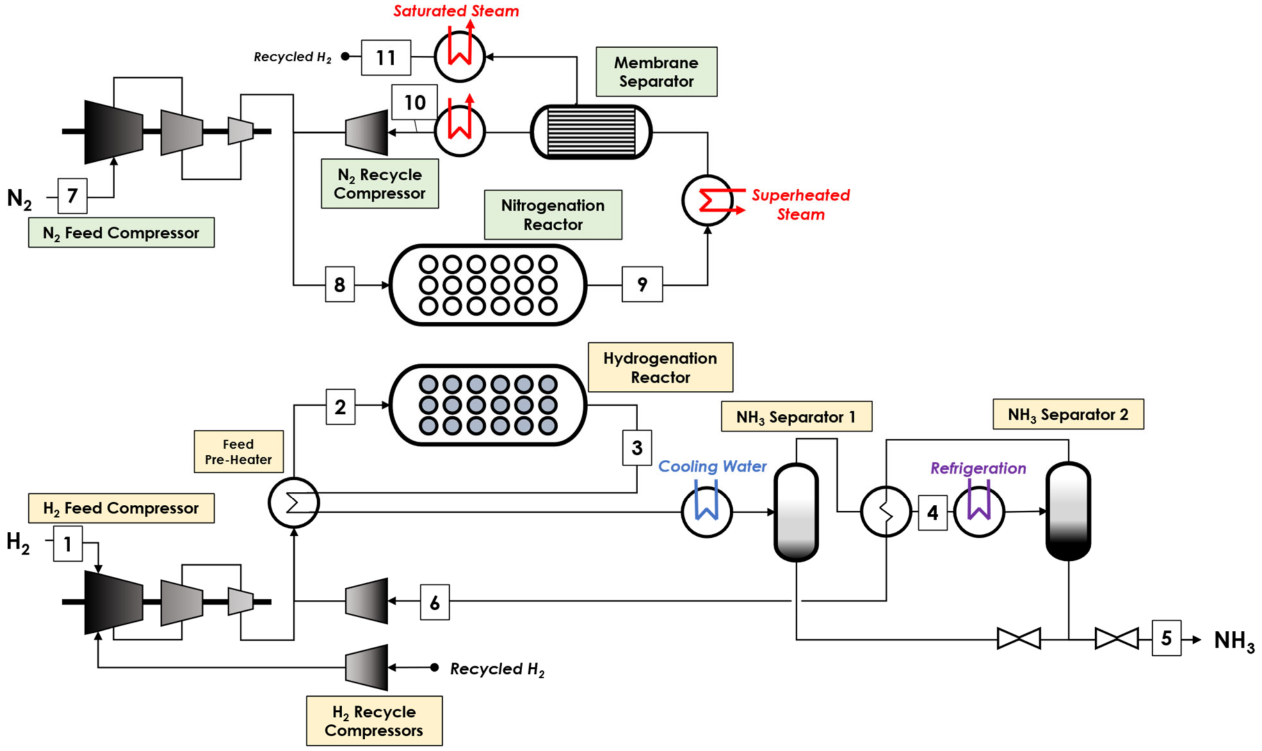

Figure S5 Process flow diagram for the chemical looping ammonia synthesis unit.

Table S7 Mass balance for the chemical looping ammonia synthesis unit. The balance for the base case (yield = 28%) is shown.

#ID	P	T	N	m	MW	Composition (% mol)		
	**bar**	**°C**	**kmol/s**	**kg/s**	**kg/kmol**	**NH_3**	**N_2**	**H_2**
1	25.0	25.0	1.50	3.02	2.02			100.0
2	149.7	250.0	7.72	16.7	2.17	1.1		99.0
3	145.2	327.6	6.72	29.7	4.42	16.0		84.0
4	140.0	13.7	6.40	24.2	3.79	11.8		88.2
5	20.0	-7.3	1.00	17.0	17.02	99.9		0.01
6	135.1	24.5	5.72	12.7	2.22	1.4		98.6
7	25.0	25.0	0.50	13.96	28.01		100	
8	150.0	250.0	3.35	93.1	27.81		99.2	0.8
9	145.5	598.7	3.35	80.2	23.94		84.3	15.7
10	140.6	281.9	2.85	79.1	27.77		99.1	0.9
11	22.6	50.0	0.50	1.00	2.02			100.0

5. Cost Correlations and detailed equipment costs:

Table S8 List of cost correlations used to calculate equipment costs. Cost correlations obtained from [16].

Equipment	Scaling parameter	Unit	Size range	A_{ref}	C_{ref} ($M)	n	F	LM*
Ammonia Synthesis Reactor	Reactor Volume	m^3	10 – 180	100	1.3	0.4	[a]	1.54
Packed Bed Reactors	Reactor Volume	m^3	1 – 500	20	0.11	0.52	[a]	1.54
Fluidised Bed Reactors	Reactor Volume	m^3	1 – 500	12	5	0.67	-	1.54
Compressor (Low Duty)	Power	MW	0.15 – 3	1	2.85	0.65	[b]	1.505
Compressor (High Duty)	Power	MW	3 – 13	10	26	0.7	[b]	1.50
Refrigeration Unit	Refrigeration capacity	MW	0.02 – 5	1	0.8	0.77	3[c]	1.3
Membrane Unit	Gas flowrate	m^3/s	-	1	2	0.93	-	0.8
Heat Exchanger	Area	m^2	20 – 2000	100	0.07	0.71	[d]	1.96

[a] Material and pressure factor applied. Stainless steel. Pressure factor calculated as follows $f_P = 0.1778 * P(bar) \div 10 + 0.7179$. [b] Material factor applied. [c] Evaporator temperature factor applied. [d] Material and heat exchanger configuration factor applied.

Table S9 Detailed equipment costs for the reference and chemical looping ammonia synthesis units.

Equipment	Costs ($M) Ref-Case	Equipment	Costs ($M) CL-Case
Ammonia Synthesis Reactor	15.0	Chemical Looping Reactors	4.0
Feed Compressor	31.4	H_2 Feed Compressor	33.8
Recycle Compressor	4.6	H_2 Recycle Compressor	5.0
Refrigeration Unit	22.5	H_2 Tail Gas Compressor	1.0
Feed Pre-Heater	5.3	N_2 Feed Compressor	2.6
Steam Super-Heater	0.4	N_2 Recycle Compressor	1.4
Steam Evaporator	0.4	Refrigeration Unit	22.2
Gas Cooler (Water)	1.7	Membrane Unit	1.8
Gas Cooler (Cold Gas)	1.6	H_2 Pre-Heater	2.6
Gas Cooler (Refrigeration)	1.1	Steam Super-Heater	0.4
		Steam Evaporator 1	2.6
		Steam Evaporator 2	0.3
		Gas Cooler (Water)	1.4
		Gas Cooler (Cold Gas)	2.2
		Gas Cooler (Refrigeration)	1.4
**Total**	**84.0**	**Total**	**80.4**

6. Chemical looping reactor sizing

Reaction rate equations for R2a and R2b were obtained from rate data in Gao et al.[3]. Equations (9) and (10) were used to determine reaction rates at higher temperatures (>300 °C). For the fixed-bed reactor configuration, the reaction temperature (T in Kelvin) for both R2a and R2b was taken as the mean temperature of the outlets (465 °C). For the fluidised bed configuration, the reaction temperatures corresponded to the values found in the simulation. The required mass of solid reacting per hour (m_{R2a} and m_{R2b}) was calculated from the required product flowrates ($n_{H_2}$and n_{NH_3}) and reaction rates of R2a and R2b (r_{R2a} and r_{R2b}) (Eq. (9)). This mass is used to size the fluidised bed reactor. The ratio of these masses (R) is equal to the required ratio of fixed-bed reactors to operate in parallel at any given time (Eq. (10)). The ratio was converted to an improper fraction to obtain the number of fixed-bed reactors for each reaction step (N_{R2a} and N_{R2b}) and the total number of fixed-bed reactors (N) (Eq. (11)). The mass of a single fixed-bed reactor (M) is calculated using equation (12).

$$rate(R2a)\left(\frac{mmol\ H_2}{kg.hr}\right) = \exp(-5833.33 \times T^{-1} + 19.54) \tag{9}$$

$$rate(R2b)\left(\frac{mmol\ NH_3}{kg.hr}\right) = \exp(-4078.95 \times T^{-1} + 10.45) \tag{10}$$

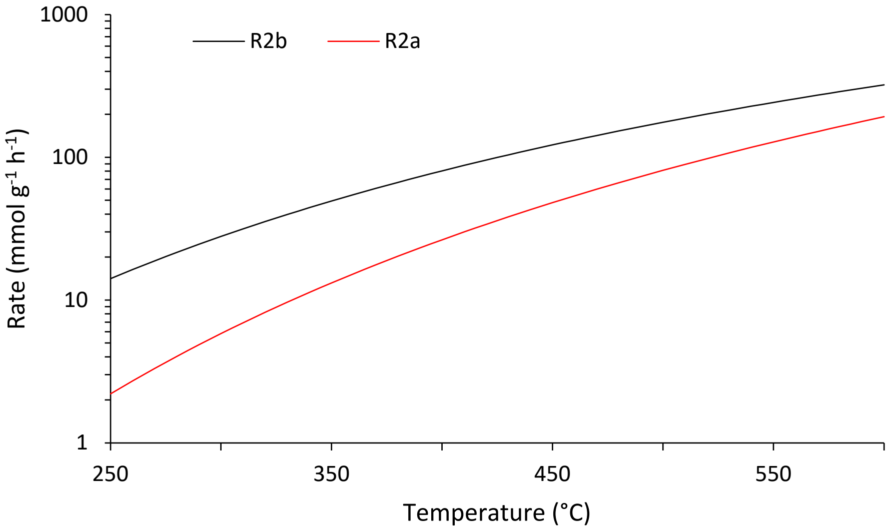

Figure S6 The reaction rates above are given in mmol of hydrogen or ammonia produced for R2a and R2b, respectively.

$$m_{R2a} = \frac{n_{H_2}}{r_{R2a}} \qquad m_{R2b} = \frac{n_{NH_3}}{r_{R2b}} \tag{11}$$

$$R = \frac{m_{R2a}}{m_{R2b}} = \frac{N_{R2a}}{N_{R2b}} \tag{12}$$

$$N = N_{R2a} + N_{R2b} \tag{13}$$

$$M = \frac{m_{R2a}}{N_{R2a}} = \frac{m_{R2b}}{N_{R2b}} \tag{14}$$

7. Vapour-liquid equilibrium of ammonia in hydrogen

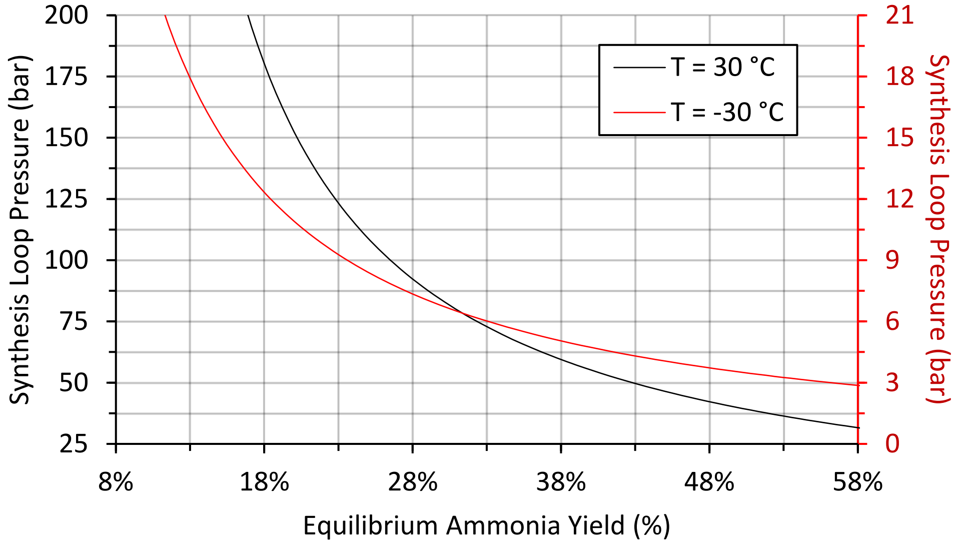

Figure S7 The synthesis loop pressure versus the equilibrium ammonia yield is shown above. The equilibrium ammonia vapour pressure can be calculated from the synthesis loop pressure and ammonia yield. The graph is meant to be a guide to determine the minimum operating pressures required to separate a given ammonia yield at 30 °C (Cooling water) or -30 °C (Refrigeration).

8. Material property uncertainty:

The deviation of the absolute entropy and enthalpy of formation of a list of metals and their oxides, nitrides, and hydrides have been evaluated by comparing their estimated values to values found in literature. Shown in Figure S8 and Figure S9 are the numbers or counts of compounds whose percentage error lie within the given range. The mean error range was used to find the uncertainty of the absolute entropy and enthalpy of formation of the benchmarked materials.

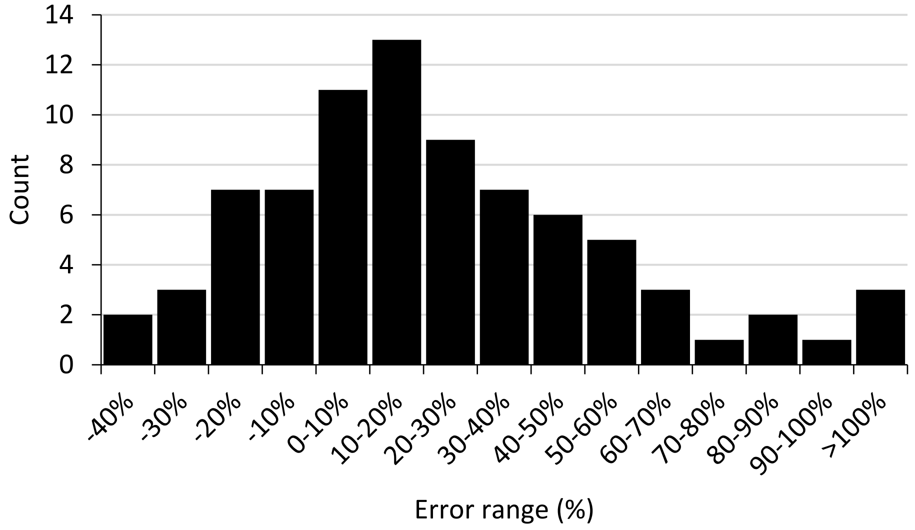

Figure S8 Distribution of percentage errors for the estimated entropies of oxides, nitrides, and hydrides.

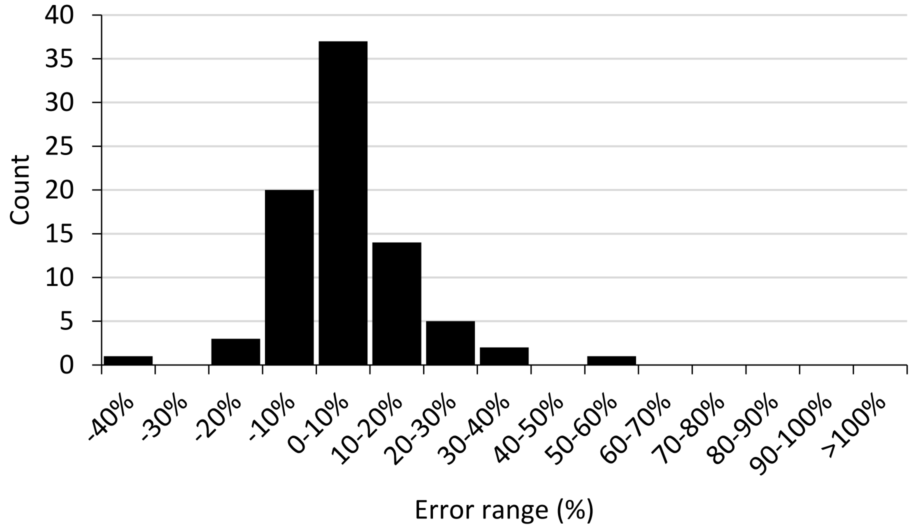

Figure S9 Distribution of percentage errors for the estimated formation enthalpies of oxides, nitrides, and hydrides.

9. Nitrogenation reaction material benchmark:

Using the same methodology provided in section 2.5 of the main text, the minimum ΔH°_{solid} and ΔS°_{solid} to achieve a specified nitrogen conversion for reaction R2a can be defined. The equilibrium constant for reaction R2a (Eq. (17)) is calculated for a specified nitrogen conversion (X_{N_2}). Shown in Figure S10 are the estimated values of ΔH°_{solid} and ΔS°_{solid} for the observed pairings in this work. Variations in pressure need not be considered since the equilibrium of reaction R2a is independent of pressure, and at 150 bar the fugacity coefficients for hydrogen and nitrogen are close to 1. The latter is considered, nonetheless. The results indicate that the hydride/imide pairs can achieve greater than 90% equilibrium conversions at temperatures ranging from 200 – 600 °C, hence the reaction is not limited by equilibrium in the simulation since a 14 – 18% conversion is achieved. High temperatures appear to favour the equilibrium conversion of reaction R2a, however this is dependent on the value of ΔS°_{solid}.

$$y_{N_2} = \left(1 - X_{N_2}\right) \tag{15}$$

$$y_{H_2} = X_{N_2} \tag{16}$$

$$K_{eq} = \frac{f_{H_2}^{0.5}}{f_{N_2}^{0.5}} = \frac{\left(y_{H_2}\phi_{H_2}P\right)^{0.5}}{\left(y_{N_2}\phi_{N_2}P\right)^{0.5}} = \frac{\left(y_{H_2}\phi_{H_2}\right)^{0.5}}{\left(y_{N_2}\phi_{N_2}\right)^{0.5}} \tag{17}$$

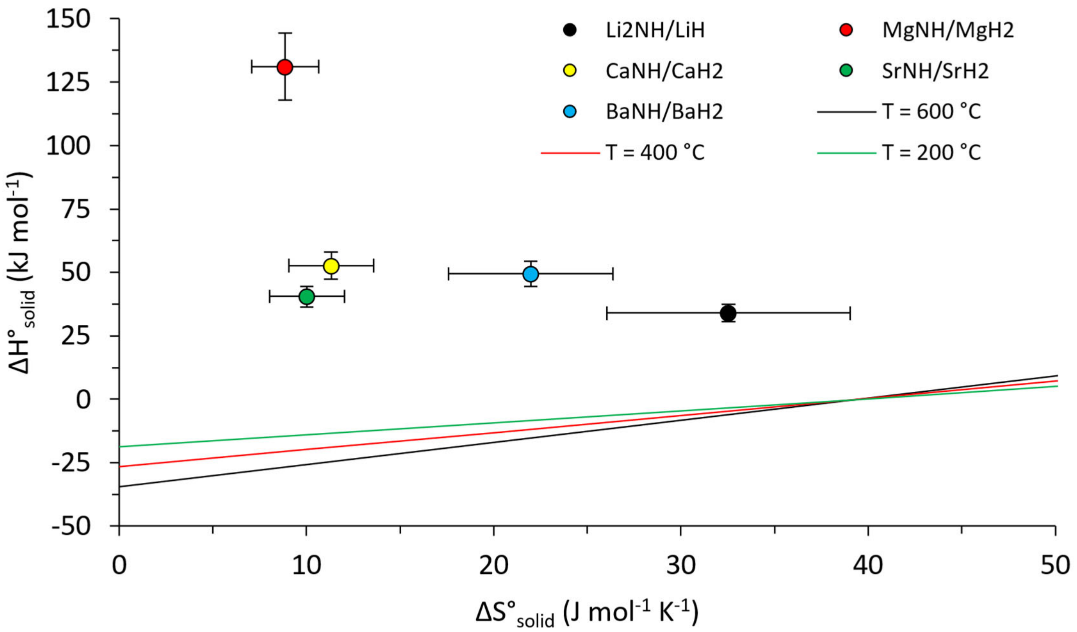

Figure S10 The minimum enthalpy and entropy change of the solid phase reaction to achieve a 90% nitrogen conversion is represented by the plotted lines. Pairings situated above or to the left these lines have properties enabling them to achieve an equal or greater conversion than what's specified. Three temperatures are considered here at 150 bar.

Supplemental references

(1) Yan, H.; Gao, W.; Wang, Q.; Guan, Y.; Feng, S.; Wu, H.; Guo, Q.; Cao, H.; Guo, J.; Chen, P. Lithium Palladium Hydride Promotes Chemical Looping Ammonia Synthesis Mediated by Lithium Imide and Hydride. *J. Phys. Chem. C* **2021**, acs.jpcc.1c01230. https://doi.org/10.1021/acs.jpcc.1c01230.

(2) Feng, S.; Gao, W.; Wang, Q.; Guan, Y.; Yan, H.; Wu, H.; Cao, H.; Guo, J.; Chen, P. A Multi-Functional Composite Nitrogen Carrier for Ammonia Production via a Chemical Looping Route. *J. Mater. Chem. A* **2021**. https://doi.org/10.1039/d0ta10519h.

(3) Gao, W.; Guo, J.; Wang, P.; Wang, Q.; Chang, F.; Pei, Q.; Zhang, W.; Liu, L.; Chen, P. Production of Ammonia via a Chemical Looping Process Based on Metal Imides as Nitrogen Carriers. *Nat. Energy* **2018**, *3* (12), 1067–1075. https://doi.org/10.1038/s41560-018-0268-z.

(4) Goto, Y.; Daisley, A.; Hargreaves, J. S. J. Towards Anti-Perovskite Nitrides as Potential Nitrogen Storage Materials for Chemical Looping Ammonia Production: Reduction of Co_3ZnN, Ni_3ZnN, Co_3InN and Ni_3InN under Hydrogen. *Catal. Today* **2020**, 364, 196-201. https://doi.org/10.1016/j.cattod.2020.03.022.

(5) Kitano, M.; Inoue, Y.; Sasase, M.; Kishida, K.; Kobayashi, Y.; Nishiyama, K.; Tada, T.; Kawamura, S.; Yokoyama, T.; Hara, M.; Hosono, H. Self-Organized Ruthenium-Barium Core-Shell Nanoparticles on a Mesoporous Calcium Amide Matrix for Efficient Low-Temperature Ammonia Synthesis. *Angew. Chemie Int. Ed.* **2018**, *57* (10), 2648–2652. https://doi.org/10.1002/anie.201712398.

(6) Wang, P.; Chang, F.; Gao, W.; Guo, J.; Wu, G.; He, T.; Chen, P. Breaking Scaling Relations to Achieve Low-Temperature Ammonia Synthesis through LiH-Mediated Nitrogen Transfer and Hydrogenation. *Nat. Chem.* **2017**, *9* (1), 64–70. https://doi.org/10.1038/nchem.2595.

(7) Gao, W.; Wang, P.; Guo, J.; Chang, F.; He, T.; Wang, Q.; Wu, G.; Chen, P. Barium Hydride-Mediated Nitrogen Transfer and Hydrogenation for Ammonia Synthesis: A Case Study of Cobalt. *ACS Catal.* **2017**, *7* (5), 3654–3661. https://doi.org/10.1021/acscatal.7b00284.

(8) Laassiri, S.; Zeinalipour-Yazdi, C. D.; Catlow, C. R. A.; Hargreaves, J. S. J. The Potential of Manganese Nitride Based Materials as Nitrogen Transfer Reagents for Nitrogen Chemical Looping. *Appl. Catal. B Environ.* **2018**, *223*, 60–66. https://doi.org/10.1016/j.apcatb.2017.04.073.

(9) Kojima, R.; Aika, K. I. Cobalt Molybdenum Bimetallic Nitride Catalysts for Ammonia Synthesis: Part 1. Preparation and Characterization. *Appl. Catal. A Gen.* **2001**, *215* (1–2), 149–160. https://doi.org/10.1016/S0926-860X(01)00529-4.

(10) Aspen Technology .inc. Aspen Plus Ammonia Model. **2008**. https://user.eng.umd.edu/~nsw/chbe446/Ammonia-Aspen.pdf

(11) Lee Pereira, R. J.; Argyris, P. A.; Spallina, V. A Comparative Study on Clean Ammonia Production Using Chemical Looping Based Technology. *Appl. Energy* **2020**, *280*, 115874. https://doi.org/10.1016/j.apenergy.2020.115874.

(12) Spallina, V.; Motamedi, G.; Gallucci, F.; van Sint Annaland, M. Techno-Economic Assessment of an Integrated High Pressure Chemical-Looping Process with Packed-Bed Reactors in Large Scale Hydrogen and Methanol Production. *Int. J. Greenh. Gas Control* **2019**, *88*, 71–84. https://doi.org/10.1016/j.ijggc.2019.05.026.

(13) Ouadha, A.; En-nacer, M.; Adjlout, L.; Imine, O. Exergy Analysis of a Two-Stage Refrigeration Cycle Using Two Natural Substitutes of HCFC22. *Int. J. Exergy* **2005**, *2* (1), 14–30.

https://doi.org/10.1504/IJEX.2005.006430.

(14) Emerson, S. C.; Emerson, S. C.; Magdefrau, N. J.; She, Y.; Thibaud-erkey, C. Advanced Palladium Membrane Scale-up for Hydrogen Separation. **2013**. https://www.osti.gov/servlets/purl/1063878.

(15) Sinnott, R. K.; Towler, G. *Chemical Engineering Design*; **2013**. https://doi.org/10.1016/C2009-0-61216-2.

(16) Woods, D. R. *Rules of Thumb in Engineering Practice*; Wiley-VCH, **2007**.
